# Tourniquet use in total knee arthroplasty and the risk of infection: a meta-analysis of randomised controlled trials

**DOI:** 10.1186/s40634-022-00485-9

**Published:** 2022-07-01

**Authors:** A. A. Magan, O. Dunseath, P. Armonis, A. Fontalis, B. Kayani, F. S. Haddad

**Affiliations:** 1 Department of Trauma and Orthopaedic Surgery, University College Hospital, 235 Euston Road, Fitzrovia, London, NW1 2BU UK; 2 Department of Orthopaedic Surgery, The Princess Grace Hospital, 42-52 Nottingham Pl, Marylebone, London, W1U 5NY UK; 3 University College London, Gower St, London, WC1E 6BT UK; 4 Institute of Sports, Health and Exercise, University College Hospital, 235 Euston Road, Fitzrovia, London, NW1 2BU UK

**Keywords:** Tourniquet, Total knee arthroplasty, TKA, Infection, Meta-analysis

## Abstract

**Purpose:**

The intra-operative use of tourniquets during Total Knee Arthroplasty (TKA) is common practice. The advantages of tourniquet use include decreased operating time and the creation of a bloodless visualisation field. However, tourniquet use has recently been linked with increased post-operative pain, reduced range of motion, and slower functional recovery. Importantly, there is limited evidence of the effect of tourniquet use on infection risk. The purpose of this systematic review and meta-analysis is to fill this gap in the literature by synthesising data pertaining to the association between tourniquet use and infection risk in TKA.

**Methods:**

A systematic literature search was performed on Pubmed, Embase, Cochrane and clinicaltrials.gov up to May 2021. Randomized control trials were included, comparing TKA outcomes with and without tourniquet use. The primary outcome was overall infection rate. Secondary outcomes included superficial and deep infection, skin necrosis, skin blistering, DVT rate, and transfusion rate.

**Results:**

14 RCTs with 1329 patients were included. The pooled incidence of infection in the tourniquet group (4.0%, 95% CI = 2.7–5.4) was significantly higher compared to the non-tourniquet group (2.0%, 95% CI = 1.1–3.1) with an OR of 1.9 (95% CI = 1.1–3.76, *p* = 0.03). The length of hospital stay, haemoglobin drop (0.33 95% CI =0.12–0.54), *P* = 0.002) and transfusion rates (OR of 2.7, 95%CI = 1.4–5.3, *P* = < 0.01) were higher in the tourniquet group than the non-tourniquet group. The difference in the length of inhospital stay was 0.24 days favouring the non-tourniquet group (95% CI = 0.10–0.38, *P* = < 0.01). The incidence of skin blistering (OR 2.6, 95% CI = 0.7–9.9, *p* = 0.17), skin necrosis (OR 3.0, 95% CI = 0.50–19.3, *p* = 0.25), and DVT rates (OR 1.5, 95% CI = 0.60–3.60, *p* = 0.36) did not differ between the two groups.

**Conclusion:**

Quantitative synthesis of the data suggested tourniquet use was associated with an increased overall risk of infection, intraoperative blood loss, need for blood transfusion and longer hospital stay. Findings of this meta-analysis do not support the routine use of tourniquet in TKA and arthroplasty surgeons should consider any potential additional risks associated with its use.

**Level of evidence:**

meta-analysis, Level II.

## Introduction

Although the use of tourniquets in battlefields dates back to The Middle Ages, the use of a pneumatic tourniquet in the operating room is credited to the famous neurosurgeon Harvey Cushing in 1904. Since then, surgeons have used tourniquets to reduce blood loss and thus create a bloodless visualization field [1]. Nowadays, tourniquet use is common practice in orthopaedics during extremity surgery [2–11]. In particular, the tourniquet has been extensively used during total knee arthroplasty (TKA), with over 90% of surgeons in the UK and USA routinely employing it for TKAs [1]. Given more than 111,000 TKAs were performed in 2019 across the UK alone, determining whether this is the optimal method is of paramount importance to ensure that the best available care is provided to patients undergoing TKA [12, 13].

The advantages of tourniquet use include reduced operative time and decreased intraoperative blood loss, which facilitates enhanced visualization of the operative field and theoretically allows for a more robust bone-cement integration [1, 14–17]. More recently, tourniquet use has also been linked with better antibiotic delivery through the intraosseous regional administration [18]. Despite its proposed benefits, however, tourniquet use in TKA has become debatable [14]. Given the significant advancements in surgical techniques, implants, and anaesthesia over the last century, TKAs may successfully be performed without the use of the tourniquet [19]. Furthermore, concerns have been raised in relation to its association with increased intraoperative and post-operative pain, reduced range of motion, reperfusion injury, slower functional recovery, increased risk of wound and skin complications and deep venous thrombosis [14, 16, 20–24]. Notwithstanding, existing evidence of the effect of tourniquet use on infection risk is limited, as there is no study to date that has investigated infection risk as a primary outcome. There is a paucity in the literature and benefits of tourniquet use should be balanced against its potential risks to reach an informed and evidence-based decision. This systematic review and meta-analysis aims to evaluate the risk of infection and other complications in TKA with and without tourniquet use.

## Materials and methods

### Eligibility criteria

**Fig. 1:**
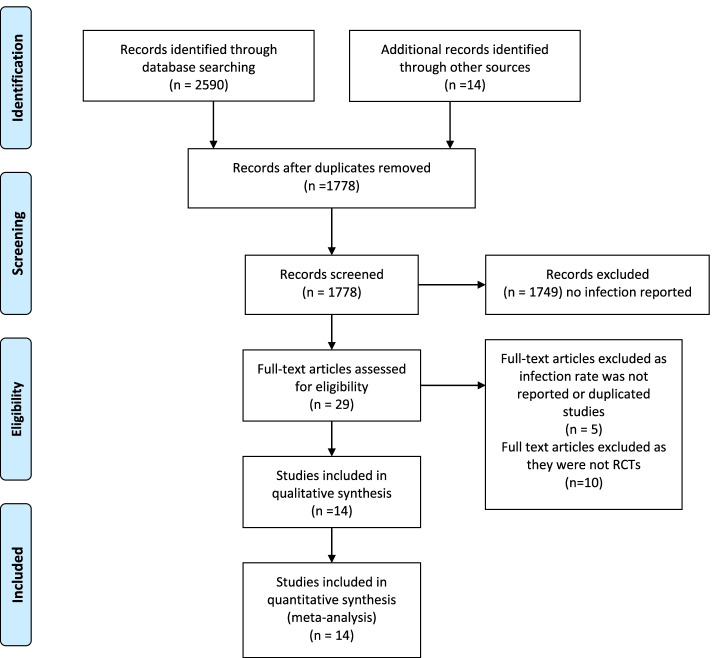
PRISMA Flowchart

The study was conducted using the Preferred Reporting items for Systematic Reviews and Meta-analysis (PRISMA) Fig. 1. The study protocol was published online at the PROSPERO international prospective register of systematic reviews and meta-analysis CR42020187902.

Eligible study designs were randomised controlled trials owing to their higher methodological rigour. We included studies encompassing patients undergoing total knee arthroplasty with or without a tourniquet and our primary outcome was rate of infection. Inconsistencies and disagreements between the two independent reviewers were resolved by reaching a consensus decision. The inclusion and exclusion criteria are detailed in Table 1.

**Table 1:** Eligibility criteria

Inclusion Criteria	Exclusion Criteria
• Randomized Controlled Trials • Studies that clearly reported infection	• Case reports • Review articles • Articles that do not report infection • Level III - level V studies

### Search strategy

A systematic literature search was performed on Pubmed, Embase, Cochrane and clinicaltrials.gov for trials published from inception to May 2021. We combined Medical Subject Headings (MeSH) with free text searching. The search terms used were “tourniquet, total knee replacement, total knee arthroplasty, infection”.

### Methodological study assessment and assessment of publication bias

**Fig. 2:**
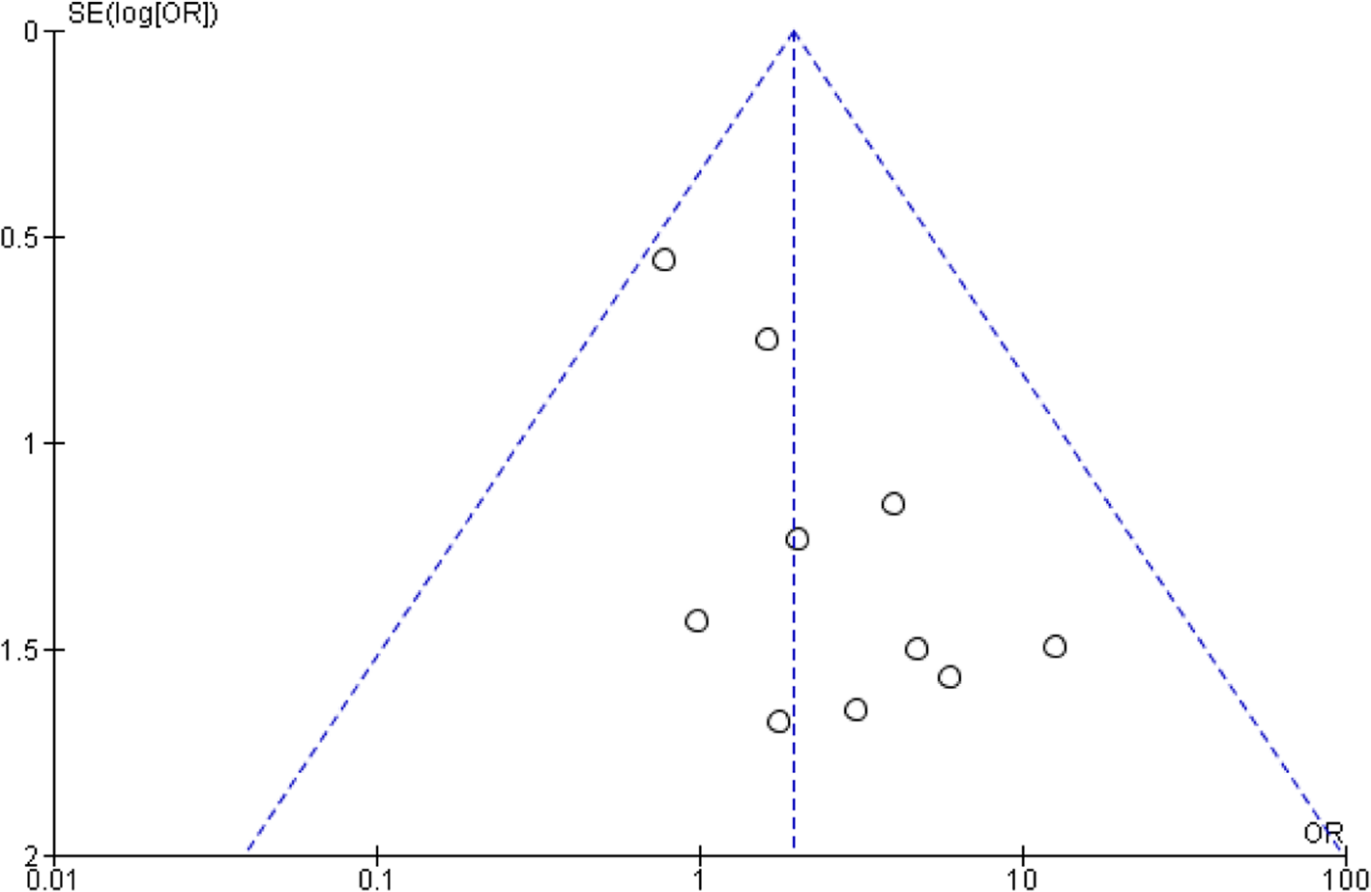
Funnel plots depicting the heterogeneity amongst studies in reporting the rate of total infection rates amongst the studies

The Cochrane Collaboration’s ‘Risk of bias’ tool was utilised to evaluate risk of bias in RCTs. The domains evaluated by the tool are: random sequence generation and allocation concealment (selection bias), incomplete outcome data (attrition bias), blinding (performance and detection bias), selective reporting (reporting bias) and other sources of bias. The quality of studies was assessed by two individual investigators. Publication bias was assessed by funnel plots looking at the effect estimate of the intervention against each study’s sample size (Fig. 2).

### Outcomes

Our primary outcome was infection rate in patients undergoing TKA with or without a tourniquet. Secondary outcomes included the following: 1) superficial infection, 2) deep infection, 3) skin necrosis, 4) skin blisters, 5) Deep venous thrombosis (DVT) rate, 6) transfusion rate, 7) postoperative Hb drop, and 7) length of hospital stay.

### Data extraction and collection

Eligible studies were independently screened by two reviewers and data was collected based on a pre-piloted standardised extraction sheet. Data collected included patients’ characteristics and demographics (age, weight or BMI, gender), number of infections (total of superficial and deep), the incidence of skin necrosis and blistering (these variables were treated as independent), deep venous thrombosis (DVT), volume of blood loss, drop in post-operative Hb and transfusion rate. For continuous variables the mean and SD (or Standard error) were recorded and when these were absent the range, median and *p* values were recorded.

### Statistical analysis

We utilised pooled odds ratios (ORs) and 95% confidence intervals (CIs) for.

all dichotomous outcomes using the Mantel–Haenszel method. For continuous outcomes we used the inverse variance method to calculate the mean difference and 95% CI.

We also assessed the heterogeneity among studies with the Chi-square test using.

Cochran’s Q statistic. We also employed the I^2^ measure to evaluate the extent; we considered heterogeneity as low if I^2^ = 25–49%, moderate if I^2^ = 50–74% and high if I^2^.

≥75%). If low heterogeneity was noted, we used the fixed-effects models to analyse our data. We used the Review Manager software version 5.4.1 for performing all the analyses.

## Results

### Studies identified

14 RCTs met the inclusion criteria and documented rate of infections (Fig. 1)

**Fig. 3:**
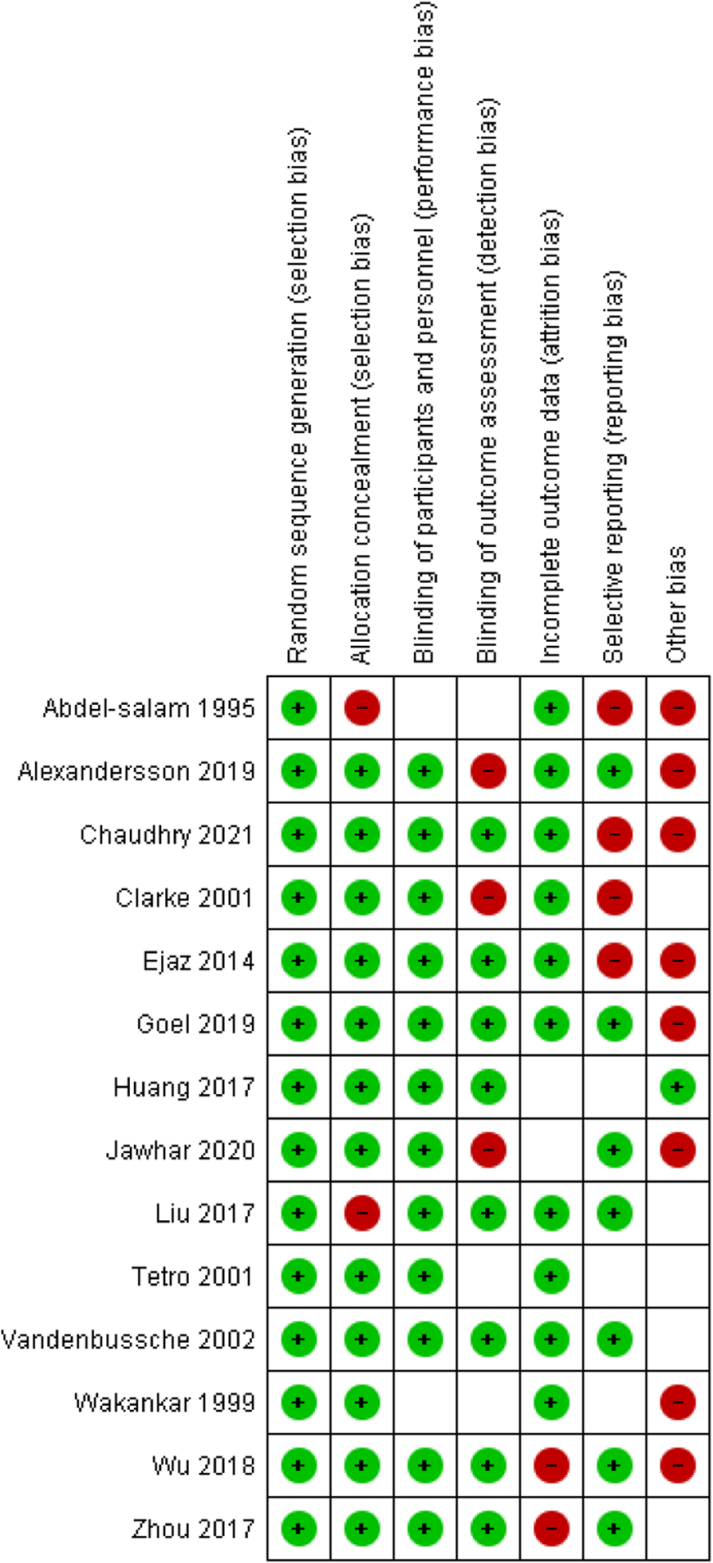
Methodological assessment of the included RCTs

Following confirmation from the authors, two RCTs were excluded from this meta-analysis as the same cohort of patients was analysed [25, 26]. Methodological assessment of the studies is depicted in Fig. 3.

### Patients

A total of 1457 patients (1509 knees - some studies included patients undergoing bilateral total knee arthroplasty) were included in this analysis. The age, BMI and gender distribution were comparable between groups (Table 2).

**Table 2:** Comparison of baseline characteristics for the two groups

		Tourniquet group	No tourniquet group	Weighted Mean difference (95%CI)	***p***	
**Total number of patients**		782	727			
**Pooled age mean (SD)**		67.2 (8.4)	67.8 (8.2)	−0.60 (−1.44–0.25)	0.17	
**Pooled BMI (SD)**		28.0 (4.4)	28.4 (4.6)	0.32 (−0.84–0.20)	0.22	
**Gender**	Male n(%)	274 (38.5%)	261 (43.7%)		0.06*	
	Female n(%)	438 (61.5%)	336 (56.3%)			

*Fisher’s exact test

### Infection

**Fig. 4:**
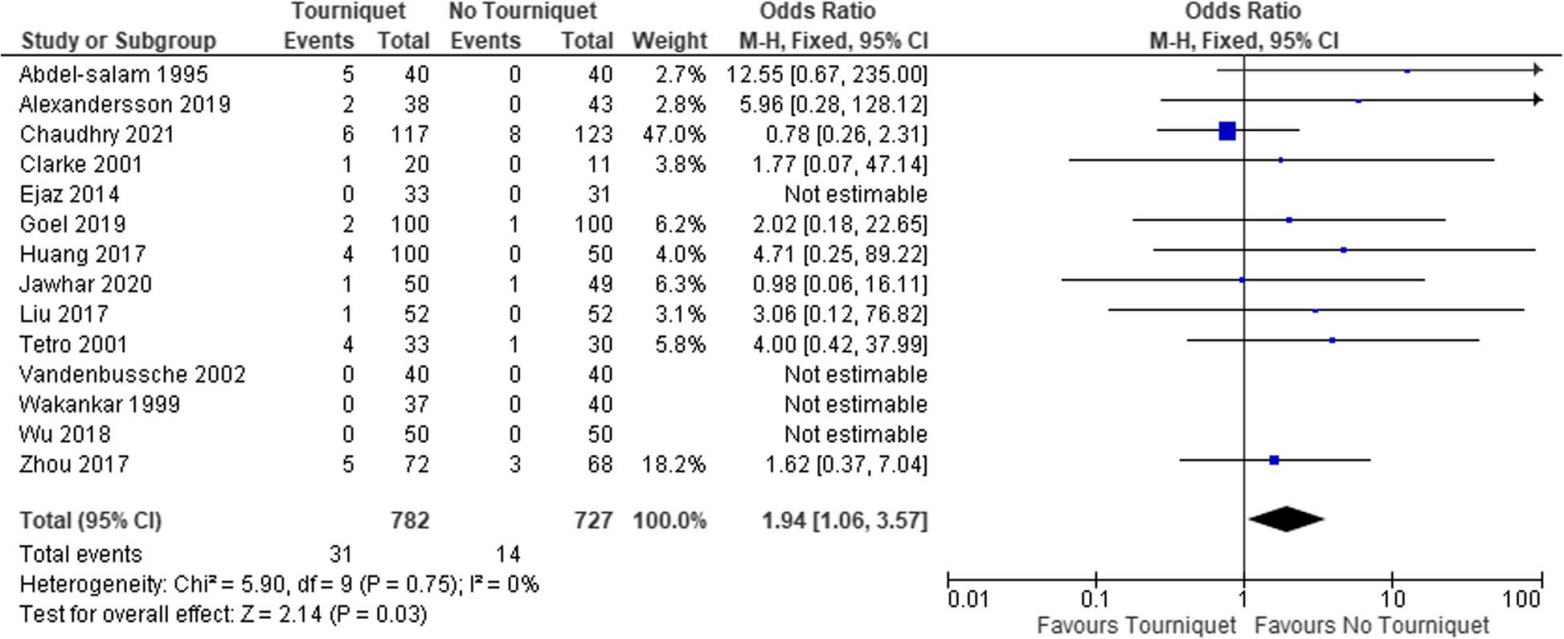
Comparison of the total infection rate (superficial and deep) between groups

There were 31 (4%) reported cases of infection (superficial and deep) in the tourniquet group, compared to 14 (2%)in the non-tourniquet group (Table 3, Fig. 4). This gave an overall pooled proportion of infection in both groups of 4.0% (95 CI = 2.7–5.4) and 2.0% (95 CI = 1.1–3.1) respectively (Table 4). Quantitative synthesis showed the difference to be significant OR 1.9 (95%CI 1.1–3.6), p 0.03, Table 4. In studies separately reporting superficial and deep infections, (10 studies), subgroup analyses revealed both were higher in the tourniquet group but this did not reach statistical significance (Table 4).

**Table 3:** Studies included with total number of cases and infections in each group

	Total Cohort(n)	Tourniquet Group n(%)	No Tourniquet n(%)	Total infection in tourniquet group n(%)	Total infection in No tourniquet group n(%)	Age (mean ± SD) in tourniquet group	Age (mean ± SD) in No tourniquet group	M/F in tourniquet group %	M/F in No tourniquet group %	Follow up length
Chaudhry et al. 2021 [27]	240	117	123	6 (5.1)	8 (6.5)	62.29 ± 9.63	65.41 ± 9.042	43.4/56.6	46.7/53.3	6 months
Zhou et al. 2017 [28]	140	72 (51.4%)	68 (48.6%)	5 (6.9)	3 (4.4)	66.8 ± 8.6	69.1 ± 7.6	18.06/81.94	10.29/89.71	6 months
Wu et al. 2018 [29]	100	50 (50%)	50 (50%)	0	0	67.58 ± 4.61	68.06 ± 3.16	44/56	38/62	6 months
Vandenbussche et al. 2002 [30]	80	40 (50%)	40 (50%)	0	0	72.5 (38–89)	68.5 (50–81)	22.5/77.5	40/60	3 months
Ejaz et al. 2014 [31]	64	33 (51.6%)	31 (48.4%)	0	0	68 ± 8.4	68 ± 7.8	54.55/45.45	54.84/45.16	1 year
Jawhar et al. 2020 [32, 33]	99	50 (50.5%)	49 (49.5%)	1 (2)	1 (2)	69.3 ± 7.4	68.3 ± 7.8	34/66	38.78/61.22	6 months
Goel et 2019 [34]	200	100 (50%)	100 (50%)	2 (2)	1 (1)	66.0 ± 7.0	65.5 ± 7.8	50/50	48/52	6–8 months
Alexandersson et al. 2019 [35]	81	38 (46.9%)	43 (53.1%)	2 (5.3)	0	68.0 ± 7.4	69.7 ± 6.4	47.37/52.63	51.16/48.84	3 months
Huang et al. 2017 [36]	150	50	50	4 (8)	0	66.2 ± 8.3	65.1 ± 6.8	36/64	32/68	6 months
Liu et al. 2017 [26, 37]	52 (bilateral knee)	52	52	1 (2)	0	67.0 ± 8.0	67.0 ± 8.0	30.77/69.23	30.77/69.23	25 months (19–36)
Tetro et al. 2001 [38]	63	33	30	4 (11.6)	1 (3.3)	69.8 ± 6.7	69.8 ± 9.0	45.45/54.55	36.67/63.33	7 days
Clarke et al. 2001 [39]	31	20	11	1 (5)	0	Not reported	Not reported	Not reported	Not reported	7 days
Wakankar et al. 1998	77	37	40	0	0	72.5 (57–85)	71.8 (43–91)	29.73/70.27	35/65	4 months
Abdel-salam et al. 1995 [20]	80	40	40	5 (12.5)	0	72 (65–80)	74 (64–82)	42.5/57.5	37.5/62.5	2 years
**Total**	1457	732	727	31	14	n/a	n/a	n/a	n/a	n/a

**Table 4:** Meta-analysis comparing the outcomes between the two groups

Outcome	Number of studies with data	Pooled proportion T (95%CI)	Pooled proportion NT (95%CI)	Meta-analysis OR (95%CI)	***P***	Heterogeneity I^**2**^ (***p***)
**Total infection**	14	4.0% (2.7–5.4)	2.0 (1.1–3.1)	1.9 (1.1–3.6)	0.03	0% (0.75)
Superficial infection	10	3.7 (2.2–5.5)	1.8 (0.8–3.1)	2.0 [0.9–4.1)	0.08	3% (0.40)
Deep infection	10	0.7 (0.2–1.6)	0 (0–1.3)	3.3 [0.3–32.5)	0.31	0% (0.96)
**Skin necrosis**	4	2.3 (0.6–5.0)	0 (0–2.2)	3.0 (0.5–19.3)	0.25	0% (1.00)
**Skin blisters**	6	4.9 (2.8–7.5)	1.9 (0.6–3.8)	2.6 (0.7–9.9)	0.17	0% (0.91)
**DVT**	11	2.2 (1.2–3.6)	1.5 (0.7–2.7)	1.5 (0.6–3.6)	0.36	0% (0.71)
**Transfusion**	7	9.5 (6.8–12.7)	3.9 (2.1–6.4)	2.7 (1.4–5.3)	< 0.01	0% (*P* = 0.76)

*CI* Confidence interval, *OR* Odd ratio, *T* Tourniquet group, *NT* No tourniquet group

### Other outcomes

**Fig. 5:**
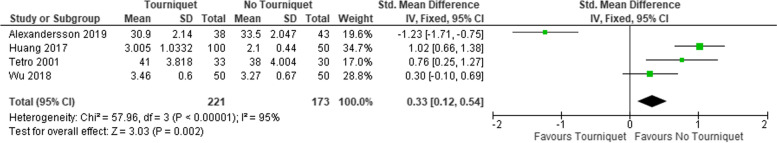
Post op HB drop between the two groups

**Fig. 6:**
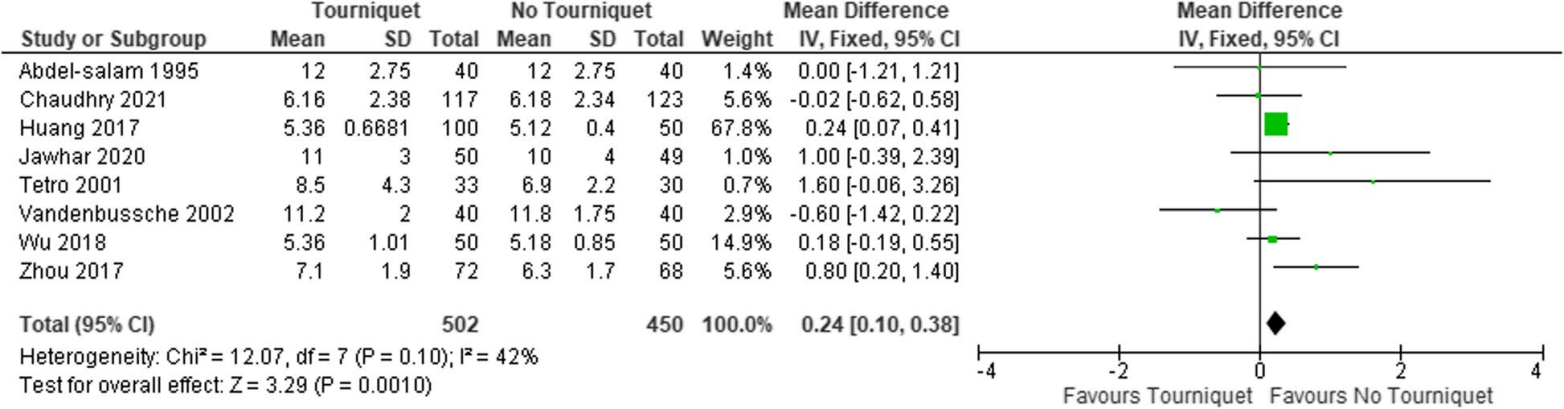
Length of hospital stay in days

**Fig. 7:**
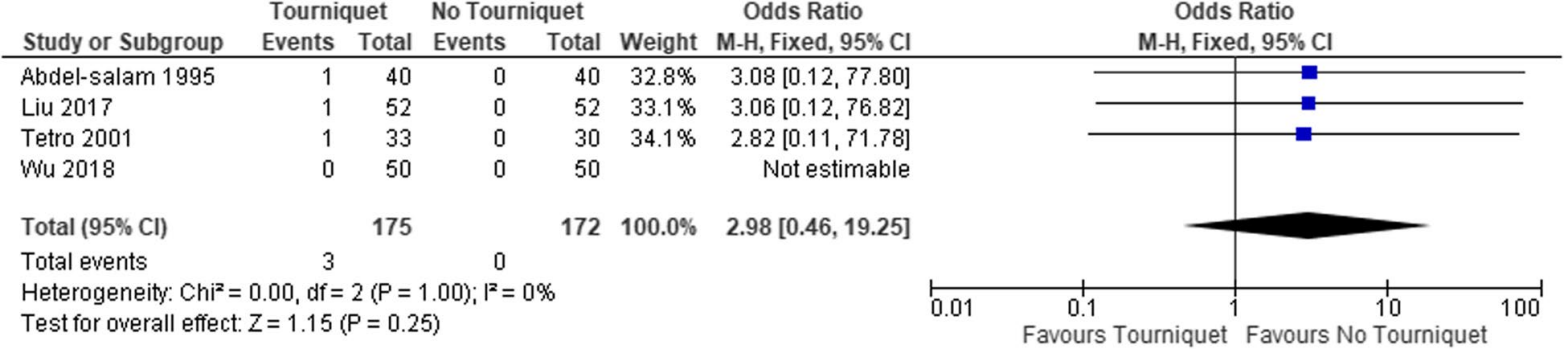
Comparison of skin necrosis between the 2 groups

**Fig. 8:**
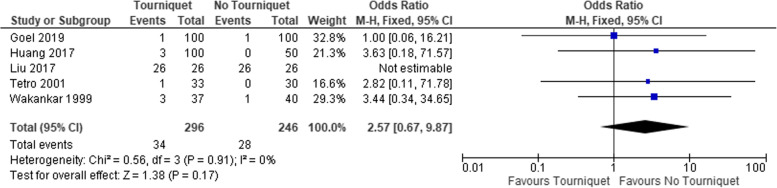
Comparing the proportion of skin blistering

The rate of transfusion (Table 4), and HB drop was also significantly higher in the tourniquet group, Fig. 5. The difference in the length of in-hospital stay was 0.24 days shorter in the non-tourniquet group (95% CI, 0.10–0.38), Fig. 6. The proportion of skin necrosis, blistering and DVT were higher in the tourniquet group, however this was not statistically significant (Table 4, Figs. 7 and 8).

## Discussion

The main finding in this study was that a pooled analysis of 14 RCTs, demonstrated that tourniquet use was associated with increased risk of post-operative infection, increased blood loss, higher transfusion rates and longer hospital stay. Amid a lack of high-quality evidence, orthopaedic surgeons are often confronted with clinical uncertainty with respect to using a tourniquet in TKA. Our systematic review and meta-analysis combined data across studies to compare clinical outcomes with or without tourniquet use. There was minimal inter- and intra-study variation regarding the incidence of infection, with heterogeneity of 0% between the included studies. Additionally, a higher incidence of skin necrosis, blistering and DVT were evident in the tourniquet group, however these differences did not reach statistical significance.

The use of the tourniquet has been considered standard of care in TKA [2, 3, 24, 40–42]. However, a growing body of evidence has brought its routine use into question [14, 19, 43]. Tourniquet use results in lengthy periods of compression and circulatory stasis, which could conceptually lead to wound complications including infection [15]. This meta-analysis synthesising data from 14 RCTs revealed a significant increase (OR, 1.9, 95 CI = 1.1–3.6) in the incidence of post-operative infection when a tourniquet was used intra-operatively, however subgroup analysis of superficial and deep infections showed comparable results. In concordance, a recent Cochrane systematic review found tourniquet use to be associated with significantly higher risk of developing wound infection (RR 2.72, 95% CI 1.15 to 6.42); however this study did not employ separate subgroup analyses of superficial and deep infections [14]. Moreover, an increased incidence of serious adverse events with tourniquet use has also been suggested by other studies in total knee arthroplasty and lower limb trauma surgery [44, 45].

Tetro et al. reported four superficial wound infections in a group of 33 patients whose TKA was performed using a tourniquet, compared to one superficial infection seen in the non-tourniquet group (*N* = 30) [38]. In another prospective, randomised controlled trial, two out of 38 patients in the tourniquet group developed an infection post-TKA; one superficial and one deep while no infection occurred in the non-tourniquet group [35]. Liu et al. performed bilateral primary TKAs on 52 patients, using the tourniquet on just one knee [37]. The study found that TKA with tourniquet use was associated with increased risk of skin necrosis and deep wound infection.

Individually, the sample sizes in these studies were too small to detect a statistically significant difference in rare complications, such as infection. In our study, the overall incidence of infection demonstrated a statistical significant difference between the groups; and comparable results within subgroup analysis of superficial and deep infections. A possible explanation could encompass the exsanguination of the limb itself during tourniquet inflation, rendering the wound edges hypoxic during the early post-operative period. As a consequence, the cellular response to wound healing is inhibited, potentially contributing to the increased number of post-operative wound complications seen with tourniquet use [46, 47]. Clarke et al. demonstrated a relationship between higher tourniquet inflation pressures and post-operative wound hypoxia [39]. The study reported one infection in the tourniquet group as opposed to none in the non-tourniquet group, suggesting tourniquet associated hypoxia may influence wound healing. However, the three arms of this study (tourniquet use at low pressure, high pressure and no tourniquet use) may have introduced confounders and bias, making the findings more difficult to interpret.

The study by Abdel-Salam et al. reported one episode of skin necrosis and four episodes of superficial wound infections in a cohort of 40 patients undergoing TKA with a tourniquet, while no wound complications were noted in the non-tourniquet group [20]. Notably, this study reports the highest percentage of infections within the tourniquet group, 12.5%, and is the oldest study included in the meta-analysis. However, the funnel plot looking at the heterogeneity of the studies reporting on the total rate of infections (Fig. 2) did not identify this study as an outlier; hence it was included in out quantitative synthesis as it fulfilled our inclusion criteria.

The presence of persistent wound oozing post-operatively has been shown to increase the risk of infection [48, 49]. Liu et al. reported increased incidence of oozing with the use of a tourniquet in a cohort of 56 patients undergoing bilateral TKA [37]. Despite the small sample size, these findings provide further insight into the impact of tourniquet use on wound healing post-TKA.

Five out of the fourteen papers included reported on the incidence of skin blistering post-operatively [34, 36–38, 50]. Pooled analysis identified no significant difference in the incidence of skin blisters when the patient was operated with the use of a tourniquet.

Eleven RCTs in our meta-analysis reported on the incidence of DVT with or without a tourniquet [20, 28–32, 34, 36–38, 50]. Ten studies reported regimes for DVT prevention, including chemical and mechanical thromboprophylaxis [20, 28–31, 34, 36–38, 50]; Furthermore, three studies reported a venous doppler ultrasound and/or Duplex ultrasonography to screen for asymptomatic DVTs [29, 36, 50]. A higher incidence of DVTs in the tourniquet group was reported in five RCTs [20, 28, 31, 32, 50]. This is also supported by a recent Cochrane systematic review [15] and a separate meta-analysis [51], both reporting a significant increase in the incidence of DVTs in patients undergoing TKA with a tourniquet. In discordance, Vandenbussche et al. reported two episodes of DVT in the non-tourniquet group compared to one patient in the tourniquet group [30]. Similarly, Goel et al. reported one post-operative DVT in the non-tourniquet group [34].

Functional outcomes were reported in eight studies [20, 27, 28, 31, 32, 34, 36, 37]. Several different scoring systems were used; three studies used the Hospital for Special Surgery (HSS) system [20, 28, 36], two studies used the Knee Injury and Osteoarthritis Outcome Score (KOOS) [31, 34], one study used the Knee Society Score (KSS) [37], and Jawhar et al. and Chaudhry et al. used the Oxford Knee Score (OKS) to measure functional outcomes [27, 32]. Despite the different scoring systems, similar scales are used for each system, with a higher score indicating a better functional outcome. Overall, no significant difference in functional outcomes was reported in seven of the studies [20, 27, 28, 32, 34, 36, 37], which is in concordance with the literature [15].

Nine studies in this meta-analysis looked at the effect of tourniquet use on the post-operative range of movement (ROM) [20, 27–29, 31, 34, 36, 37, 50], out of which six reported a significant difference in ROM favouring surgery without a tourniquet [20, 28, 29, 31, 36, 50]. Conversely, Liu et al. and Chaudhry et al. documented no difference between cohorts [27, 37]. Of note, the population included in the studies performed by Liu et al. and Chaudhry et al. had pre-existing osteoarthritis [27, 37]. Overall, results demonstrate a trend towards faster recovery in relation to ROM postoperatively without the use of tourniquet, which may improve patient satisfaction [36].

Twelve studies [20, 27–32, 34–37, 50] reported pain-related outcomes; seven of which found significantly reduced pain in patients undergoing surgery without a tourniquet [20, 28–31, 36, 37]. In detail, in six of the above studies differences in pain severity were noted early in the first 24 hours following surgery [20, 28–30, 36, 37]. The explanation for increased pain post-operatively with tourniquet use may be that the increased mechanical compression disrupted blood circulation and led to more muscle ischaemia [33, 52–54].

The reduction in blood loss and optimisation of the operative field have historically been the main reasons for using a tourniquet in TKA [19]. Vandenbussche et al., Goel et al. and Chaudhry et al. reported significantly increased blood loss in patients operated without a tourniquet [27, 30, 34]. However, six studies in this meta-analysis reported no significant differences [20, 28, 29, 31, 36, 38]. One explanation for these conflicting results is that the reduction of intra-operative blood loss with the use of a tourniquet could have been offset by blood loss secondary to tourniquet induced ischemia [38, 55]. Findings of a RCT reported that patients in the non-tourniquet group had increased intraoperative blood loss (215.7 ± 113.7 ml vs 138.6 ± 93.9 ml, *p* < 0.001), notwithstanding post-operative blood loss and drain output were reduced [28]. Wu et al. also reported no statistically significant difference in total blood loss with or without a tourniquet (1039.86 ± 251. 98 ml vs 1103.95 ± 201.93, *p* = 0.614) [29]. Our meta-analysis showed a significant reduction in post-op Hb, suggesting the use of a tourniquet may not in fact confer the expected benefits.

Our meta-analysis has several limitations. Firstly, included RCTs reported infection as a secondary outcome. There is a risk that follow-up was insufficient for the manifestation of infections, potentially resulting in delayed deep infections not being captured. Discrepancies between each paper in the inclusion criteria may also have introduced bias. Selection bias could also be present as some papers solely included patients with osteoarthritis [27, 29, 35–37, 39], whereas others encompassed both osteoarthritis and rheumatoid arthritis patients [20, 38]. Finally, a few studies had several domains characterised as unclear or high risk utilising the Cochrane Collaboration’s Risk of bias tool; hence reflecting potential bias that may have been introduced.

## Conclusion

This meta-analysis of RCTs suggested that tourniquet use in TKA was associated with an increased overall risk of infection, intraoperative blood loss, need for blood transfusion and longer hospital stay. Subgroup analyses encompassing superficial and deep infections as an outcome revealed a non-statistically significant trend favouring non tourniquet use. Results of our meta-analysis do not justify the routine use of tourniquet in TKA and arthroplasty surgeons should be considerate of the potential additional risks involved.
